# Revisited Hyperoxia Pathophysiology in the Perioperative Setting: A Narrative Review

**DOI:** 10.3389/fmed.2021.689450

**Published:** 2021-10-22

**Authors:** Stefano Busani, Marco Sarti, Francesco Serra, Roberta Gelmini, Sophie Venturelli, Elena Munari, Massimo Girardis

**Affiliations:** ^1^Cattedra e Servizio di Anestesia e Rianimazione, Azienda Universitaria Policlinico di Modena, Modena, Italy; ^2^Chirurgia Generale d'Urgenza e Oncologica, Azienda Universitaria Policlinico di Modena, Modena, Italy

**Keywords:** hyperoxia, surgical patient, critically ill, wound repair, pathophysiology, reactive oxygen species

## Abstract

The widespread use of high-dose oxygen, to avoid perioperative hypoxemia along with WHO-recommended intraoperative hyperoxia to reduce surgical site infections, is an established clinical practice. However, growing pathophysiological evidence has demonstrated that hyperoxia exerts deleterious effects on many organs, mainly mediated by reactive oxygen species. The purpose of this narrative review was to present the pathophysiology of perioperative hyperoxia on surgical wound healing, on systemic macro and microcirculation, on the lungs, heart, brain, kidneys, gut, coagulation, and infections. We reported here that a high systemic oxygen supply could induce oxidative stress with inflammation, vasoconstriction, impaired microcirculation, activation of hemostasis, acute and chronic lung injury, coronary blood flow disturbances, cerebral ischemia, surgical anastomosis impairment, gut dysbiosis, and altered antibiotics susceptibility. Clinical studies have provided rather conflicting results on the definitions and outcomes of hyperoxic patients, often not speculating on the biological basis of their results, while this review highlighted what happens when supranormal PaO_2_ values are reached in the surgical setting. Based on the assumptions analyzed in this study, we may suggest that the maintenance of PaO_2_ within physiological ranges, avoiding unnecessary oxygen administration, may be the basis for good clinical practice.

## Introduction

Oxygen has been a cornerstone of medical care for decades, but also one of the most misused remedies. Maintaining proper homeostasis between oxygen demand and delivery is crucial. Oxygen administration has become an established practice in hospital daycare, although the evidence in favor of its use is rather uncertain. The perioperative period is one of the phases in which the administration of oxygen reaches its greatest use. The exposure to transient post-operative hypoxemia caused by pain, excessive sedation, or other reasons, often justified an even ill-considered use of this medical gas. An additional reason why anesthesiologists tend to administer high oxygen flows in the perioperative period is based on the World Health Organization (WHO) guidelines, which recommend an 80% of inspired oxygen (FiO_2_) fraction to reduce infections of the surgical site (SSIs) in adult patients undergoing general anesthesia with tracheal intubation (1). Although the WHO recommendations from 2016 to 2018 were downgraded from strong to conditional, the general recommendation to ventilate surgery patients with 80% FiO_2_ was still maintained (2). Alongside this recommendation, the relative safety of high inspired FiO_2_ in the surgical patient has favored its massive use (3). The antimicrobial properties of oxygen are due to the bactericidal properties associated with increased reactive oxygen species (ROS) production and were already recognized in the 1980s - “oxygen as an antibiotic” (4), but recently other oxygen-related mechanisms have been suggested playing a role in wound repair (5).

On the contrary, recent randomized controlled trials (RCTs) conducted in different clinical settings have shown that higher oxygen delivery negatively affects the outcome of patients (6–8) while other studies have not shown such negative effects (9, 10). Hence, a great debate has arisen on the need to administer a high FiO_2_ in critically ill, post-operated, or cardiac arrest patients. The discussion in the scientific community focused mainly on clinical data deriving from RCTs, but these data hardly provided a pathophysiological explanation of the mechanism of action of oxygen during hyperoxia.

Therefore, we aimed to perform a narrative review on the pathophysiology of high oxygen administration in patients undergoing surgery, focusing on the effects that hyperoxia has on surgical wound healing and the systemic macro and microcirculation, lungs, heart, brain, kidneys, gut, coagulation, and infections.

## Methods

The pathophysiological evidence for hyperoxia, reported here, was found through searches in the Medline (PubMed) and Scopus databases without time limits. We have outlined the pathophysiological mechanisms, using alternatively the following keywords “hyperoxia” and “surgical wound”; “hyperoxia” and “macrocirculation”; “hyperoxia” and “microcirculation”; “hyperoxia” and “lung” or “pulmonary circulation”; “hyperoxia” and “heart”; “hyperoxia” and “brain”; “hyperoxia” and “kidneys” or “erythropoiesis”; “hyperoxia” and “gut” or “bacterial translocation”; “hyperoxia” and “surgical anastomosis”; “hyperoxia” and “coagulation”; “hyperoxia” and “perioperative infections.” The language restriction used included the acceptance of articles in English only.

This study is a narrative review, and, as such, is being relatively non-specific from a methodological point of view; it focuses on articles reporting the pathophysiological aspects of high oxygen delivery. To provide a simpler understanding to the reader, we had to shorten the available bibliographic sources, mainly by discarding papers without a pathophysiological basis, considering that the terms entered generated a total of over 7,000 results. Moreover, we discarded all the papers regarding cardiopulmonary bypass and cardiac surgery, because we wanted to focus mainly on non-cardiac surgery.

## Wound Healing

### Physiological Basis

After surgery, the wound's healing process is conditioned by multiple factors, particularly age, patient's nutritional status, comorbidities (such as renal insufficiency, oncological disease, diabetes), and adequate tissue oxygenation. SSI is a frequent complication after surgery, because of the susceptibility of the surgical wound to poor oxygen tension. For this reason, hyperoxia has been considered to reduce the SSI risk. The surgical incision causes the skin layer's interruption and subcutaneous blood vessels dissection, creating ischemia of the surrounding microenvironment. At the same time, activation of platelets, coagulative factors, growth factor release, lymphocytes', polymorphonuclear, and macrophages chemotaxis occur. In this complex network, there is a need for increased oxygen demand due to higher cells' metabolic activity (11). Furthermore, activated neutrophils convert oxygen and glucose into hydrogen ions, superoxide, and lactic acid, resulting in oxygen consumption equal to 50 times the baseline values (5). Low oxygen tension could play a role as an early stimulus for tissue repair, as fibroblasts and endothelial cells are shown to be affected by hypoxic stimuli in terms of migration, protein synthesis, and proliferation. On the other hand, hypoxia leads to poor healing and increases susceptibility to infections. Most of the available oxygen is then used to perform antimicrobial functions, collagen synthesis, angiogenesis, and epithelization (11).

### Pathological Consequences

In the first 48 h, neutrophils are the major players in innate immunity and infection prevention. Their function depends on the high partial pressure of oxygen (PO_2_), which produces ROS, activates phagocytosis and bactericidal defense against wound pathogens; moreover, as stated above, oxygen is the substrate used by phagosomal oxidase to catalyze the formation of the superoxide ion (5). Superoxide is bactericidal by itself and promotes the ability to kill bacteria by producing other ROS (5). For these reasons, hyperoxia plays a crucial role in wound healing; therefore, the whole process is strictly sensitive to the PO_2_ in the tissues (5–12) (Table 1). ROS also enhances the signal transduction of growth factors such as PDGF, VEGF, and HIF-1, which stimulates neovascularization, activation of fibroblast, and collagen deposition (11).

**Table 1:** Main pathophysiological mechanisms related to hyperoxia effects (see text for details).

**Wound healing**	• Neutrophils activation with ROS production (5) • Superoxide bactericidal effect (11) • ROS enhances the signal transduction of growth factors which stimulates neovascularization, activation of fibroblast, and collagen deposition (11)
**Macrocirculation**	• Increase of systemic vascular resistance due to reduction of NO (13, 14) • Decrease in Left ventricular end-diastolic area, Stroke Volume, Cardiac Output, Heart Rate (15–18)
**Microcirculation**	Decrease in vascular density due to: • Reduction in NO bioavailability (13) • Inactivation of potassium and calcium channels • Alterations of arachidonic acid metabolism. • Reduction of the synthesis of vasodilating prostaglandin (19) • Microthrombosis (20, 21)
**Lungs**	• Oxidative stress responsible for pulmonary cellular necrosis and apoptosis (22) • Damage-associated molecular patterns release inducing the production of inflammatory cytokines (22) • ROS as secondary messenger causing the activation of other pro-inflammatory pathways (23) •Resorption atelectasis and surfactant alteration (24, 25)
**Heart**	• Coronary blood flow reduction due to oxidative degradation of coronary endothelium-derived NO by ROS (26) • Myocardial ischemia (27–30)
**Brain**	• Contradictory theories on the activation of apoptotic cascades, neuronal inflammation, and necrosis (31) • Decreased cerebral blood flow (32)
**Kidneys**	Controversial results about the association between intraoperative hyperoxia and development of AKI during the post-operative period (33–35)
**Erythropoiesis**	• Hypoxia is the trigger for erytropoietin production (35) • “Normobaric oxygen paradox”: a short time exposure to normobaric hyperoxia followed by a rapid return to normoxic condition leads to a “relative hypoxia” which stimulates erytropoietin production (36)
**Gut and bacterial traslocation**	• “Gut-Lung Axis”: their bacterial communities are correlated, and gut microbiota protect the host during pneumonia (37) • Oxidative stress impairs bacterial structure (37) • Increase of Gram-Negative Enterobacteriaceae recognized by TLR-4, involved in necrotizing enterocolitis and in lung injury (38)

*Legenda: ROS, reactive oxygen species; NO, nitric oxide; AKI, acute kidney injury*.

The PROXI randomized clinical trial investigated the effect of hyperoxia on SSIs within 14 days of surgery; the authors concluded that there were no significant differences between the patients receiving a FiO_2_ of 80% compared to the group that received a Fi0_2_ of 30%. This conclusion is still debated because of several study limits, i.e., to consider in the same group elective and emergency surgery (44). On the contrary, both Belda et al. (45) that Greif et al. (46) reported a lower rate of SSI in patients treated with hyperoxia.

## Cardiovascular

### Physiological Basis

The cardiovascular effects of hyperoxia are conflicting. The main confounder in several studies seemed to be the time of hyperoxia exposure (47). Anyway, however, the unanimously recognized effect of the enhanced oxidative stress is the systemic vascular resistances (SVR) increase, with no modifications observed in norepinephrine serum levels (47). The increase of SVR seems to be dose-dependent when blood oxygen level overcomes the physiological range, so there is a linear dose-response relationship between arterial oxygen and cardiovascular parameters in supranormal conditions (48). This phenomenon may be mediated by the direct effect on vessels depending on the interplay between oxygen and the reduction of the bioavailability of nitric oxide (NO), an intrinsic vasodilator, due to serum level increase in ROS (13, 14) (Table 1). This effect on the vessels has been observed mainly in the skeletal muscles, which are the main determinant of SVR. Moreover, a recent experimental report highlighted a decrease in endothelial cell viability and proliferation under hyperoxia conditions (49).

### Pathological Consequences

Hyperoxia has been observed to increase cardiac parasympathetic activity as well as arterio-cardiac baroreceptor reflexes suggesting that peripheral vasoconstriction is the first event following this neural adaptations (50). Another effect shown by Bak et al. (48) consists of left ventricular end-diastolic area (LVEDA) reduction after the exposition to supraphysiological oxygen levels: this event occurred without heart rate (HR) reduction suggesting a venous return and central blood volume decrease. Secondary to these hemodynamic alterations, this study showed also a reduction in stroke volume (SV) and cardiac output (CO). On the contrary, Bodetoft et al. (15) through a magnetic resonance imaging study reported that LVEDA did not modify. A decrease in SV and CO was observed in a RCT on ten healthy volunteers by Yoann Gole et al. (16) during hyperoxia; after the end of hyperoxic exposure SV and CO normalization occurred later than HR normalization. A dose-dependent effect of hyperoxia was also observed in the reduction of HR. This, associated with the absence of significative variations of SV, looks like the principal determinant of the reduction of CO observed in hyperoxic conditions (15–18) (Table 1).

## Microcirculation

### Physiological Basis

The effects of hyperoxia on microcirculation are manifold and have been described on various vascular beds in the perioperative period. The sublingual vascular bed, thanks to its easy accessibility, was the one that received the most attention. Experimental studies on sublingual microvasculature have shown that with PaO_2_ increasing there was a progressive decrease in the vascular density (19, 20). The drop in microcirculatory perfusion was not associated with cellular hypoxia (e.g., the lactate value remained constant), but by an increase in SVR which was explained by multiple mechanisms: reduction in NO bioavailability due to an increase in ROS, inactivation of potassium and calcium channels, alterations of arachidonic acid metabolism, a decrease of cyclooxygenases activity that led to a reduction of the synthesis of vasodilating prostaglandin (19) (Table 1). Waisman et al. demonstrated that hyperoxia administration after splanchnic induced ischemia had beneficial effects by attenuation of both local and remote inflammatory microvascular responses (21).

### Pathological Consequences

Smit et al. (51), confirming the previous mechanisms, have added that arteriolar and venular microthrombosis occurred after hyperoxia exposure (Table 1). This underlined the correlation between the high level of PaO_2_ and the activation of hemostasis. Microcirculation alterations become relevant during anemia, where hyperoxia compromises the compensatory vasodilation of the microcirculation inducing a decrease of tissue oxygen delivery and PO_2_, despite an increase of arterial oxygen content (52). Mechanisms of skin vasoconstriction related to hyperoxia are still unclear, but several theories have been proposed. Yamazaki et al. showed that this may depend on the vasodilating prostaglandins inhibition and the decrease of NO bioavailability caused by ROS (13).

## Lung and Pulmonary Circulation

### Physiological Basis

ROS are the main responsible for histological pulmonary modifications. Their levels increase proportionally to PaO_2_. The main source of ROS is presented by oxidative phosphorylation but also secondary to the stimulation of microbes, cytokines, and xenobiotics. When the ROS levels overcome those of the antioxidant defenses appears a condition of oxidative stress responsible for the damage to fats, proteins, and nucleic acids that can bring pulmonary cellular necrosis and apoptosis (22). The ROS-induced damages also determine damage-associated molecular patterns release that induces the production of inflammatory cytokines by the neutrophils (Table 1). This response can be amplified to the point of generating a systemic inflammatory response syndrome (22). The inflammasome pattern mediated by ROS also provides for the activation of an alternative Toll-like receptors (TLRs) pathway culminating in the activation of the transcription factor nuclear factor-kappaB (NF-kb), which controls the expression of an array of inflammatory cytokine genes (53). Huang et al. (54) demonstrated, with an *in vitro* study, that intracellular ROS could be an endogenous ligand for TLR2/4 determining the production of pro-inflammatory cytokines like IL-6 and IL-8. NF-kb pathways also seem to regulate the expression of other inflammatory cytokines like TNF-alpha, MIP-2, PA-1, IL-1beta. Those are essentials for the first phases of the inflammatory response, while an increase of HMGB-1 seems to burden the late inflammatory phase exacerbating the lung injury (55). ROS also acts like a secondary messenger causing the activation of other pro-inflammatory pathways. One of these is the MAPK pathway that has many effects on cellular growth, proliferation, differentiation, stress response, survival, and apoptosis (56). Many MAPK intracellular cascades have been described: ERK1/2, JNK, p38, ERK5 activation. The effects change due to the cell type involved and duration of exposition (57).

The development of pulmonary circulation is a long and complex process regulated by several transcription factors and growth factors during prenatal, as well as postnatal period, in which many events could happen and alter this process. In particular, the organs development takes place in a hypoxic environment, the uterus. Preterm newborns pass from this condition to a relative hyperoxic postnatal condition (room air). Moreover, they are usually undergone to mechanical ventilation with high dose of oxygen to promote the maturation of different organs. Hyperoxia and ventilation injury cause oxidative stress and inflammatory response leading to a damage of the lung vasculature, with common complications of premature babies, represented by persistent pulmonary hypertension of infants and bronchopulmonary dysplasia. Hyperoxia and ROS cause a degradation of the alpha subunit of the transcriptional hypoxia-inducible factor 1 and the reduction of vascular endothelial growth factor expression, consequently. This process interrupts angiogenesis and alveolarization (23).

It is known that the dysregulation of pulmonary vascular development during the perinatal period leads to pulmonary vascular disease in adulthood (58). This has been well-studied in mice with echocardiography and cardiac magnetic resonance: mice exposed to hyperoxia were characterized by signs of pulmonary hypertension and right ventricular (RV) dysfunction, like higher RV pressure, decreased RV stroke volume and low ejection fractions (59).

### Pathological Consequences

The lung is the first organ involved by the toxic effects of high oxygen concentrations during and after surgery. The pulmonary oxygen toxicity presents two sequential phases (60). In the first acute step, interstitial and alveolar edema formation was observed, due to the pneumocytes type I loss, lymphatic vessels distention, and inflammatory cells infiltration. The onset of this condition is dose-dependent related to FiO_2_ levels: this pathological feature was historically described in tracheobronchitis and progressive alveolar edema, but a full regression of these lesions was described (61) after the discontinuation of oxygen administration. The second phase, called proliferative, could be defined as time-dependent; it was observed in the case of persistence over time of the oxygen administration. This was characterized by the proliferation of type II pneumocytes (which replace pneumocytes type I) causing enlargement of the alveolar epithelial basement membrane, fibroblastic infiltration appearance, hyaline membrane formation, macrophagic infiltration appearance, and alveolar volume (60, 61) reduction. In patients who survive after prolonged exposition to oxygen, emphysema, atelectasis, bronchiectasis, and interstitial fibrosis were found. Those changes, that appeared after the proliferative phase, were not reversible after the oxygen administration withdrawal. The effects that have been found are like those observed in patients with acute respiratory distress syndrome and/or ventilator-induced lung injury (62). Besides inflammatory effects, other lung tissue alterations were also observed during high FiO_2_ administration. Resorption atelectasis is secondary to the rapid passage of oxygen from alveoli to capillary blood, with an increased incidence of postoperative pulmonary and infective complications (24). Moreover, hyperoxia also leads to surfactant metabolism and turnover alteration with an increase of the alveolar collapse risk and compliance impairment (24, 25) (Table 1).

The respiratory mechanics is also affected by hyperoxia in the perioperative period. During spontaneous breathing after a short period of hypoventilation, mediated by chemoreceptors in the carotid bodies, an increase of the minute ventilation secondary to a rise of tidal volume occurred (63). These changes are not related to an elevation of the respiratory rate. This response to hyperoxia is observed also in patients with denervation of carotid bodies, so it is more likely to be secondary to a central mechanism (e.g., neurons of the solitary complex sensible to hyperoxia were found in mice) (63).

## Heart

### Physiological Basis

As mentioned above, hyperoxia is associated with increased vascular resistance in the small arteries like the coronary ones, resulting in coronary blood flow (CBF) reduction and increased myocardial oxygen consumption. However, the administration of supplemental oxygen to patients with coronary heart disease is a common practice based on the idea of helping the ischemic myocardium, mainly in the post-operative phase. The arterial oxygen concentration is an important determinant of coronary artery tone and hypoxia has been described as a strong vasodilator (26).

Moreover, hemoglobin and myocytes may also play a role in the regulation of microvascular resistance during hyperoxia. Hemoglobin acts like a NO-mediated vasodilator in a deoxygenated environment, while in a high-oxygenated one it loses this role. Myocytes produce adenosine, which reduces vascular resistance and increases blood flow in a low-oxygen situation. In a context of hyperoxia, however, myocytes release angiotensin I, which brings to the production of endothelin by the endothelial cells, leading to vasoconstriction (27). The relation between hyperoxia and cardiac lactate production is controversial.

### Pathological Consequences

McNulty et al. (28) studied 18 patients with stable coronary heart disease who underwent 100% oxygen by face mask for 15 min, showing that the administration of pure oxygen increased coronary resistance by 40% and decreased CBF by 30%, with a higher concentration of NO metabolites in the coronary vessels. The vasoactive oxygen effect could be explained by the fact that hyperoxia may accelerate the oxidative degradation of coronary endothelium-derived NO by ROS (Table 1). Since ROS can have several adverse effects on ischemic myocardium, it is important to remember that supplemental oxygen administration to patients with coronary artery disease may increase myocardial ischemia and worsen myocardial ischemia-reperfusion injury (29). As adjuvant therapy, the administration of the antioxidant vitamin C could protect against the reduction in CBF velocity related to high-concentration oxygen therapy (30).

Guensch et al. (27) demonstrated that the administration of pure oxygen to patients with the multivessel coronary artery disease (CAD) was associated with lactate production, due to the reduction of CBF that increased myocardial ischemia. Moreover, they demonstrated that 14 of the 25 CAD patients (56%), hyperoxia induced post stenotic myocardial deoxygenation with a subsequent oxygenation discordance across the myocardium. These alterations were accompanied both with cardiac index and ejection fraction reduction (27).

## Brain

The impact of hyperoxia on the brain has been extensively studied in patients with cardiac arrest (64), however, this narrative review falls outside this clinical context.

### Physiological Basis

Excessive supplementation of oxygen could aggravate a primary or secondary brain injury because of the free radical production and the activation of apoptotic cascades (31), but in some preclinical models these considerations have been completely refuted and even a neuroprotective role was attributed to hyperoxic conditions (32) (Table 1). A delicate balance exists in the brain between the useful and harmful effects of oxidative stress which involves several elements (unsaturated lipid enrichment, glucose, mitochondria, calcium, glutamate, RNA oxidation, etc.) to perform different signaling functions (65).

### Pathological Consequences

A clinical model studied on the role of hyperoxia is that of cerebral ischemia, which can represent one of the most fearful perioperative complications. A retrospective multicenter cohort study demonstrated an independent correlation between hyperoxia and higher in-hospital mortality in ventilated stroke patients. They defined hyperoxia as PaO_2_ > 300 mmHg, hypoxia as PaO_2_ <60 mmHg or PaO_2_/FiO_2_ <300 mmHg, while the other cases were considered normoxia (31). Moreover, in healthy people hyperoxia was associated with an 11–33% reduction of cerebral blood flow, as well as during neurological damage, as excessive oxygen induces a reduction of oxygen delivery, worsening the injury. For this reason, the guidelines in critically neurologic patients indicate a target saturation of 94–98% to maintain normoxia (66).

The administration of supplemental oxygen is also a common clinical practice during the management of patients with subarachnoid hemorrhage (SAH). As mentioned above, oxidative stress due to hyperoxia is associated with neuronal inflammation and necrosis and with decreased cerebral blood flow. After a subarachnoid hemorrhage, hyperoxia could increase the risk of developing delayed cerebral ischemia, which is a serious complication after a SAH, raising the mortality and morbidity for survivors. An observational cohort study showed that exposure to hyperoxia was associated with a three-time-higher risk of delayed cerebral ischemia and twice the risk of poor 3-month-neurological outcome in patients with subarachnoid hemorrhage who required mechanical ventilation (67). Again Reynolds et al., in a single-center retrospective cohort study, demonstrated that, after SAH, early hyperoxia was independently associated with the occurrence of vasospasm but not with mortality (68). For this reason, in daily clinical practice, it is necessary to bear in mind that exposure to hyperoxia can have an impact on morbidity in patients after SAH.

## Kidneys

### Physiological Basis

Preterm infants, who are usually exposed to supplemental oxygen therapy that induces oxidative stress, might have impairment of organs development, like kidneys, due to this hyperoxic condition. The results of different studies are controversial. A pre-clinical study on mice exposed to a normoxic environment or to a hyperoxic one (65% FiO_2_), has shown that there are no immediate and long-term adverse effects of hyperoxia on the development of glomerular structure in premature neonates (33). However, a previous study conducted on rats exposed to 80% FiO_2_, showed that neonatal hyperoxia causes long term kidney damage, involved in pathogenesis of hypertension, and reduction of nephron number (34).

### Pathological Consequences

The studies about the effects of perioperative hyperoxia on acute kidney injury (AKI) in the postoperative days are limited and controversial. A sub-analysis of a previous trial about intraoperative supplemental oxygen was conducted to study the renal outcome of patients undergone to colorectal surgery. They were divided into two groups: ones were exposed to 30% FiO_2_ during the surgery, the other one to 80% inspired intraoperative oxygen. The outcomes were AKI during the first 7 postoperative days, defined as an increase of serum creatinine > 1.5-fold or > 0.3 mg/dL, and the maximum postoperative in-hospital creatinine level. The results showed that there was no correlation between high levels of intraoperative FiO_2_ and postoperative AKI (35).

## Erythropoiesis

### Physiological Basis

It is well-known that renal tissue hypoxia represents the classic trigger for erythropoietin (EPO) production (36). In this context, the “Normobaric oxygen paradox” is a phenomenon that consists in a short time exposure to normobaric hyperoxia and in a rapid return to a normoxic condition: this causes a “relative hypoxia” that stimulates erythropoietin production, through the regulation of Hypoxia-Inducible Factor 1-alpha expression (69) (Table 1).

### Pathological Consequences

A study (69) involving healthy volunteers compared serum erythropoietin levels in normobaric and hyperbaric oxygen conditions. In the first case, people breathed normobaric 100% oxygen for 2 h and the EPO levels measured after 24 and 36 H confirmed the “Normobaric Oxygen Paradox.” In the second case, the subjects breathed hyperbaric oxygen for 1.5 h in a hyperbaric chamber, but after 24 H a 53% decrease in serum EPO was registered. The reasons are unclear, but probably a high level of peripheral oxygen pressure is maintained after hyperbaric oxygenation and this due to a major quantity of oxygen dissolved in the plasma and in the renal tissue (69).

Anemia is a common condition of critically ill intensive care patients, generally caused by inflammatory cytokines that inhibit the EPO production. A prospective observational study has examined the efficacy of the normobaric oxygen paradox in increasing EPO levels in critical patients and the effects of hyperoxia in terms of oxidative damage, circulating levels of NO, mROS, and antioxidant. The results show that a 2-h exposure to hyperoxia in intensive care patients leads to a slight increase in EPO levels in the next 48 h; a decrease in microvascular perfusion and elevated serum ROS levels are registered during hyperoxia, followed by normalization on returning to the baseline FiO_2_ (37).

## Gut and Bacterial Translocation

### Physiological Basis

The intestinal microbiota supports local mucosal immunity and represents an important modulator of the immune system, in fact we can talk about the “Gut-Lung Axis” because their bacterial communities are correlated, and gut microbiota might protect the host during pneumonia infections. The exposure to hyperoxia in mice alters both lung and gut microbial communities and this condition precedes the oxygen-induced-lung injury because oxidative stress impairs bacterial structures (38). In premature neonates, the association between hyperoxia and postnatal growth restriction, alters the microbiota of the distal small bowel and cecum, leading to an increase in pro-inflammatory Gram-negative Enterobacteriaceae: the recognition of lipopolysaccharide in the cell wall of Enterobacteriaceae by the host TLR-4 is important in the pathogenesis of necrotizing enterocolitis. TLR4 signaling is also involved in lung injury and inflammation (70). Moreover, hyperoxia might have a positive role during colon surgery. The surgery manipulation induces oxidative stress in colonic mucosa because of activation of xanthine oxidase (XO) in enterocytes.

### Pathological Consequences

In a randomized trial, patients undergone elective colon resection were divided to receiving 30% of FiO_2_ or 80% FiO_2_ during surgery, to determine whether higher oxygen levels might reduce gut oxidative stress. The results showed that the administration of 80% FiO_2_ prevented oxidative stress, with a reduction of lipid peroxidation and glutathione oxidation; this may be due to decrease in XO enzymatic activity and XO / (XO + XDH) ratio in the colonic mucosa (39).

## Dehiscence of the Surgical Anastomosis

### Physiological Basis

The prevention of dehiscence of the surgical anastomosis has been extensively studied with the use of hyperbaric oxygen therapy (HBOT). Its positive effects are the improvement of tissue oxygenation, the reduction of cytokines' storm, the neo-vascularization improvement and the stimulation of stem cells and growth factors (40). On the other hand, it is considered a low-risk therapy. In some cases, HBOT is applied after surgery or when the anastomotic dehiscence is already present; in other cases, it could be used in the preoperative period as a preconditioning treatment to prevent the complication. Anyway, there are different and controversial studies about its use. Tissue ischemia represents the main cause of anastomotic break, it is possible to obtain an adequate tissue oxygenation with HBOT (71).

### Pathological Consequences

The histopathological evaluation in preoperative and postoperative treatment on normal and ischemic colonic anastomoses in rats has shown that HBOT has positive effects especially on ischemic anastomosis when the treatment is combined before and after surgical time (71). Conversely, Fontoura-Andrade et al. have compared pre and postoperative hyperbaric oxygen therapy on anastomosis in rats, using 100% oxygen at a maximal pressure of 2.4 atmospheres for 60 min in two preoperative sessions and four postoperative ones. They concluded that the HBOT does not change the outcome of anastomosis healing (41). Nowadays, HBOT represents just a support to prevent anastomotic dehiscence.

## Coagulation

### Physiological Basis

The direct effects of hyperoxia on coagulation pathway during surgery are still unclear. However, the pro-coagulant effects of the administration of high dose of oxygen are well-known. Hyperoxic acute lung injury is characterized by a procoagulant status with high levels of plasminogen activator inhibitor-1 and thrombomodulin, and low levels of protein C both in plasma and pulmonary oedema fluid. This leads to impairment of fibrinolysis (72). The experimental treatment with murine Activated Protein C was not enough to improve lung injury (72).

### Pathological Consequences

If we consider the 28-day in-hospital mortality and new onset of organ dysfunction in patients with myocardial injury, it has been evaluated the association between hyperoxia and negative outcomes with coagulation dysfunction due mainly to a net decrease in platelets level (73).

## Perioperative Infections

As mentioned above, a positive effect of perioperative hyperoxia is represented by the prevention of surgical wounds infections, which are sites sensitive to hypoxia.

### Physiological Basis

However, it has been demonstrated that hyperoxia creates an environment rich in free ROS which impairs the antibacterial function of macrophages. This is due to an alteration of the actin cytoskeleton with changes in cell morphology, pseudopod formation and increased cell size. It has been shown that macrophages exposed to hyperoxia have trouble in phagocytose and killing of Klebsiella Pneumonia, even if the oxidative stress leads to production of NO, a free radical involved in bacterial killing (42). Pseudomonas Aeruginosa represents one of the major pathogens involved in ventilator-associated pneumonia in hyperoxic conditions. Again, the exposure to ROS causes a macrophage damage that results in compromised Pseudomonas phagocytosis during administration of high oxygen concentrations (FiO2 ≥95%) as well as during moderate hyperoxic therapy (FiO2 65%). It has been shown that the treatment with antioxidant agents preserves actin cytoskeleton and increases phagocytosis, even in hyperoxic environment (43).

### Pathological Consequences

High oxygen levels could influence antibiotic susceptibility of different pathogens because the standard antibiotic susceptibility is normally studied on bacteria growth under normal oxygen conditions. There are several conditions in which oxygen levels are low such as intra-abdominal abscess, lungs of cystic fibrosis patients or burn wounds, where the biofilm formation of pathogens like Pseudomonas Aeruginosa and Staphylococcus species is facilitated and their tolerance to antibiotics is increased. At the same time, oxygen therapy could expose pathogens to high levels of oxygen and alter their response to antibiotics. Anoxic conditions caused reduced sensitivity of bacteria to aminoglycoside antibiotics (74). Oxygen reduction causes a decrease in susceptibility of Pseudomonas Aeruginosa and Klebsiella Pneumoniae to Piperacillin/Tazobactam and Azithromycin, respectively. Conversely, high oxygen concentration decreases the power of tetracyclines to kill bacteria. For these reasons, it might be important to also evaluate the oxygen condition of the infection site to choose the antibiotics to administer during and after surgery (74).

## Conclusions

The use of oxygen at high doses to avoid post-operative hypoxemia together with suggested hyperoxia to reduce SSI are common clinical practices. However, both of these habits must be carefully counterbalanced by growing pathophysiological evidence that highlights the damage of hyperoxia on different organs. High systemic oxygen delivery may induce oxidative stress, vasoconstriction, altered microperfusion, lung injury, hemodynamic impairments, brain ischemia, surgical anastomosis impairment, gut dysbiosis, and altered antibiotics susceptibility. Clinical trials, to date, have given rather conflicting results on the definitions and outcome of hyperoxic patients; however, the pathophysiological basis of supranormal values of PaO_2_ appear deleterious, so the maintenance of PaO_2_ within physiological ranges, avoiding the administration of unnecessary oxygen, should represent one of the cornerstones of “*good medicine.”*

## Author Contributions

SB, MS, FS, and EM designed the study and wrote the paper. RG and MG reviewed the edited manuscript. SV and SB reviewed the available literature. All authors contributed to the article and approved the submitted version.

## Conflict of Interest

The authors declare that the research was conducted in the absence of any commercial or financial relationships that could be construed as a potential conflict of interest.

## Publisher's Note

All claims expressed in this article are solely those of the authors and do not necessarily represent those of their affiliated organizations, or those of the publisher, the editors and the reviewers. Any product that may be evaluated in this article, or claim that may be made by its manufacturer, is not guaranteed or endorsed by the publisher.
